# Automatic Detection of Animals in Mowing Operations Using Thermal Cameras

**DOI:** 10.3390/s120607587

**Published:** 2012-06-07

**Authors:** Kim Arild Steen, Andrés Villa-Henriksen, Ole Roland Therkildsen, Ole Green

**Affiliations:** 1 Department of Engineering, Aarhus University, Finlandsgade 22, 8200 Aarhus N, Denmark; E-Mails: andreshenriksen@gmail.com (A.V.-H.); ole.green@agrsci.dk (O.G.); 2 Department of Bioscience, Aarhus University, Grenåvej 14, 8410 Rønde, Denmark; E-Mail: oth@dmu.dk

**Keywords:** thermal imaging, image processing, human-wildlife relationship, wildlife-friendly farming

## Abstract

During the last decades, high-efficiency farming equipment has been developed in the agricultural sector. This has also included efficiency improvement of moving techniques, which include increased working speeds and widths. Therefore, the risk of wild animals being accidentally injured or killed during routine farming operations has increased dramatically over the years. In particular, the nests of ground nesting bird species like grey partridge (*Perdix perdix*) or pheasant (*Phasianus colchicus*) are vulnerable to farming operations in their breeding habitat, whereas in mammals, the natural instinct of e.g., leverets of brown hare (*Lepus europaeus*) and fawns of roe deer (*Capreolus capreolus*) to lay low and still in the vegetation to avoid predators increase their risk of being killed or injured in farming operations. Various methods and approaches have been used to reduce wildlife mortality resulting from farming operations. However, since wildlife-friendly farming often results in lower efficiency, attempts have been made to develop automatic systems capable of detecting wild animals in the crop. Here we assessed the suitability of thermal imaging in combination with digital image processing to automatically detect a chicken (*Gallus domesticus*) and a rabbit (*Oryctolagus cuniculus*) in a grassland habitat. Throughout the different test scenarios, our study animals were detected with a high precision, although the most dense grass cover reduced the detection rate. We conclude that thermal imaging and digital imaging processing may be an important tool for the improvement of wildlife-friendly farming practices in the future.

## 1. Introduction

During the last decades, strong competition in the agricultural sector has resulted in the development of high-efficiency farm equipment. This acceleration has also included efficiency improvement of moving techniques, which means that for instance grass cutting involves working speeds exceeding 15 km/h and working widths of more than 14 m. Although the extent to which wildlife populations may be affected negatively by farming operations is difficult to assess, there is no doubt that the risk of wild animals being accidentally injured or killed during routine farming operations has increased dramatically over the years.

Several species are likely to be negatively affected by mowing operations. These include not only common farmland species, but also endangered species like the corncrake (*Crex crex*) [1,2]. In particular, the nests of ground nesting bird species like grey partridge (*Perdix perdix*) or pheasant (*Phasianus colchicus*) are vulnerable to farming operations in their breeding habitat both as a result of the nests being destroyed [3] or the incubating female being killed or injured [4]. In mammals, the natural instinct of e.g., leverets of brown hare (*Lepus europaeus*) and fawns of roe deer (*Capreolus capreolus*) to lay low and still in the vegetation to avoid predators increase their risk of being killed or injured in farming operations [4]. As a result of the increase in both working speed and width, adults of otherwise mobile species, e.g., fox (*Vulpes vulpes*) and roe deer, are now at risk of being killed or injured in farming operations as they may be unable to escape the approaching machinery.

Relatively few attempts have been made to assess the extent to which farming operations may negatively affect wildlife populations. In Germany, [4] estimated that at least 84,000 roe deer fawns, 153,000 brown hares, 11,000 wild rabbits (*Oryctolagus cuniculus*), 249,000 pheasants and 69,000 grey partridges were killed in farming operations. This corresponded to 14.5, 13.4, 1.1, 22.9 and 21.9% of the annual hunting bag, respectively. In Sweden, [5] estimated that fawn mortality caused by mowing ranged from 25–44% of the yearly recruitment during a three year study. In [6] the estimated leveret losses range from 17–44% in forage and grass fields, whereas losses were much lower in arable crops, ranging from 2–4% in spring barley (*Hordeum* spp.) and winter wheat (*Triticum* spp.), respectively. In Bulgaria, [3] estimated leveret mortality to be 27% in fodder plant biotopes. In France, [7] found that harvesting operations were of minor importance in adult hares, whereas [8] found no relationship between the juvenile proportion and grass leys or whole-crop, suggesting that farming operations had no significance on recruitment in Danish hares. The above examples show that mortality resulting from farming operations may be significant, although highly variable depending on the species, age class and habitat type.

Besides the potential effects on wildlife populations, fodder contaminated with carcasses of animals may impose a health hazard for live stock from infection by the bacteria *Clostridium botulinum* causing botulism [9]. This may lead to commercial loss, which can be substantial.

Moreover, an aspect that has only received little attention is the mental stress imposed on the farmers, who occasionally face an injured animal during farming operations. The health and safety issue associated with the farmer having to do a mercy killing without the professional expertise should not be ignored.

Various methods and approaches have been used to reduce wildlife mortality resulting from farming operations. Delayed mowing date, altered mowing patterns (e.g., mowing from the center outwards [10,11]) or strategy (e.g., leaving edge strips), longer mowing intervals, reduction of speed or higher cutting height [2,10] have been suggested to reduce wildlife mortality rates. Likewise, searches with trained dogs prior to mowing may enable the farmer to remove e.g., leverets and fawns to safety, whereas areas with bird nests can be marked and avoided. Alternatively, various scaring devices such as flushing bars [10] or plastic sacks set out on poles before mowing [5] have been reported to reduce wildlife mortality.

However, wildlife-friendly farming often results in lower efficiency Therefore, attempts have been made to develop automatic systems capable of detecting wild animals in the crop without unnecessary cessation of the farming operation. For example, a detection system based on infrared sensors has been reported to reduce wildlife mortality in Germany [12]. In [13] a UAV-based system for roe deer fawn detection is presented. The authors show that thermal imaging can be used to detect roe deer fawns based on aerial footage, however the detection is still performed manually.

Here we present a novel approach based on thermal imaging, which has been widely used to detect human activity [14–17], whereas in animals, thermal imaging has been used to estimate cervid population densities in various habitats [18,19], to detect and census mammals [20], for aerial surveys of mammals [21,22], to study nighttime behaviour in grey partridges [23] and to detect migrating birds around offshore wind turbines [24]. These examples illustrate the wide range of applications of thermal imaging; however, most often the detection of both human and animal activity has been semi-automated, and therefore based on subsequent manual inspection of recorded images. In our study, we assessed the suitability of thermal imaging in combination with digital image processing to automatically detect animals present in the crop during mowing operations as part of a wildlife-friendly farming system.

## 2. Materials and Methods

### 2.1. Study Area

The experiment took place on the 27th of June 2011 in west Jutland, Denmark (WGS84: North 56°4.355′, *East*8°23.053′). The weather was partly sunny with temperatures ranging between 22–24 °C.

### 2.2. Study Animals

For our purpose, we used a domestic rabbit (*Oryctolagus cuniculus domesticus*) and a domestic chicken (*Gallus domesticus*) as study animals. The rabbit was chosen to resemble a leveret, whereas the chicken resembles a partridge or a pheasant. Specifically, we wanted to investigate whether the insulative property of feathers, which minimize the thermal differential between them and the environment [20], would hamper the detection a bird in the crop. The study animals were kept in a cage during the experiments.

### 2.3. Infrared Thermography

Infrared thermography is based on the measurement of the radiation mode of heat transfer of a body in the infrared spectrum, which is a function of the temperature of the body [25]. All matters with a temperature above 0 K emit radiation [26]. In the infrared wavelength spectrum, mid-wave infrared (MWIR: 2.5–7 *μ*m) and long-wave infrared (LWIR: 7–14 *μ*m) are the most interesting wavelengths for imaging [27].

### 2.4. Equipment

**Figure 1.**
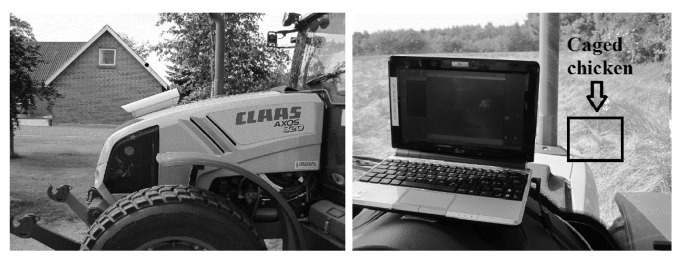
Illustration of test setup. Figure 1(left) shows the placement of the camera, and Figure 1(right) shows the inside the tractor, where the caged chicken is visible on the laptop screen.

An uncooled Forward Looking Infrared (FLIR) thermal camera was used for the recordings. The camera works in the Long-Wave Infrared Band (LWIR), which is preferred for animal detection, since the emitted radiation radiation from objects at ambient temperatures (300 K) peaks in LWIR [28]. The robustness of the camera minimizes the risk of vibrations during mowing operations. The camera was mounted (Figure 1(left)) to ensure good coverage of the area right in front of the tractor. The distance to the crop was approximately 4.75 m at an angle of approximately 75° perpendicular to the ground. The camera had a field of view of 25°, giving a working width of 2.1 m in the centre of the video frame. The recorded video was stored on a laptop, which was also used to control the settings for the camera. The box in Figure 1(right) indicates the caged chicken used in the experiment. The tractor used in the experiment was a Claas Axos 320.

### 2.5. Data Collection

The cages with the study animals were placed in the grass to imitate a natural situation, *i.e.*, the grass was ready for mowing. They were kept in the same place throughout the experiment, except for one case, where the chicken was placed in dense grass cover. The tractor was driven at different speeds (rabbit: 4, 8, 12 and 15 km/h; chicken: 5, 10 and 15 km/h) using the same track. The camera temperature range was set at 10–35 °C.

**Figure 2.**
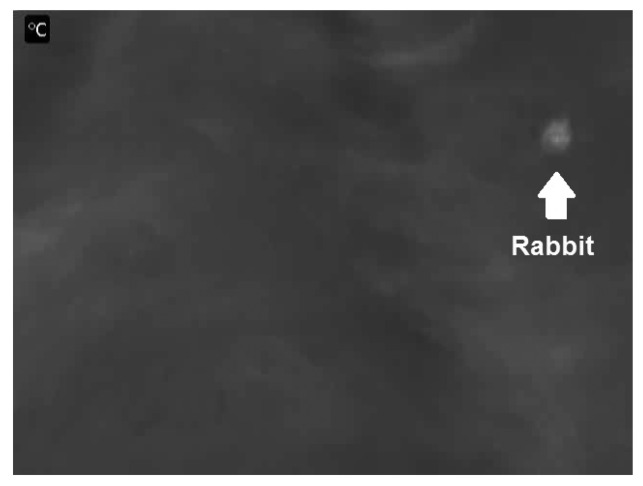
A single frame from video recording of rabbit at 4 km/h. The rabbit appear brighter than the background on the thermal image. The somewhat higher temperature in the wheel tracks can also be recognized.

When recording animals with thermal cameras, they appear brighter than the background (Figure 2) which is a result of the higher thermal radiation from the animal compared to the grass. The bright patches in the images that are not animals result from the sun's effect on the grass, which is being heated throughout the day.

### 2.6. Digital Image Processing

**Figure 3.**
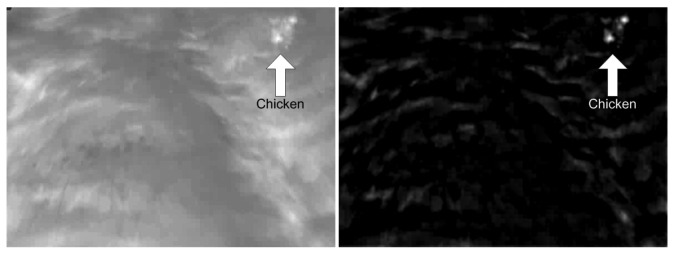
Illustration of the effect of pre-processing. Figure 3(left) is the original image of a chicken, and Figure 3(right) is the filtered image. The filtering enhances the chicken so it can be discriminated from the background.

We used digital image processing techniques in order to automatically detect our study animals on the basis of the video recordings. Ideally, the thermal radiation of the study animals exceeds the radiation from the background, which makes the animal appear brighter on the video images. However, during sunlight periods, the thermal differences between the animal and the background may become smaller and some patches of grass may radiate almost the same temperature as the animals, as in Figure 3 where grass patches appear almost as bright as the chicken. In this case filtering techniques can be applied to enhance the appearance of the animals. For this purpose, we used the Laplacian of Gaussian (LoG), also known as the Mexican hat function, filter (1) for pre-processing to enhance the appearance.

(1) $$\nabla^{2}h(r)=-\left[\frac{r^{2}-\sigma^{2}}{\sigma^{4}}\right]\text{exp}\left(-\frac{r^{2}}{2\sigma^{2}}\right)$$

Here *r*^2^ = *x*^2^ + *y*^2^, which are the x- and y-size of the filtering mask. The standard deviation controls the degree of blurring in the image. The size of the filter and blurring is based on the size of the animal, *i.e.*, the number of pixels, and in this study the parameters were *σ* = 0.5 and *x* = *y* = 50. The filter suppress the diffuse patches in the background, whereas the animal (the chicken) is enhanced (Figure 3).

**Figure 4.**
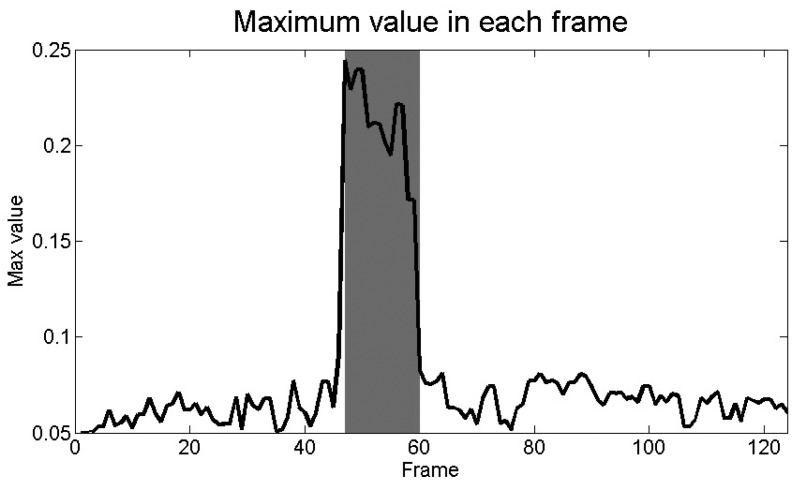
Plot of maximum values in the frames after pre-processing. The frames where an animal (the rabbit in this case) is present is marked with dark-grey. It is therefore possible to detect the presence of the animal on the basis of the maximum values in the frame.

On the basis of the pre-processed image, it is possible to identify the animal using adaptive thresholding [29], where the threshold value is based on the maximum pixel value of the current image compared to the mean value of maximum pixel values of previous images (10 images have been used in test). This is because maximum values increase significantly when an animal is present in the image (Figure 4), and this rapid increase in the values can be used to detect the animal in the video. The threshold value is therefore adaptively set with respect to the maximum pixel value within the image, when a significant increase in maximum values has been detected. When a significant decrease in maximum values is detected, the threshold value is set to a default value above the current maximum value within the image. This is performed after the LoG filter has been applied, and hot patchy areas of grass does not introduce a significant increase in maximum pixel values as the animals do.

**Figure 5.**
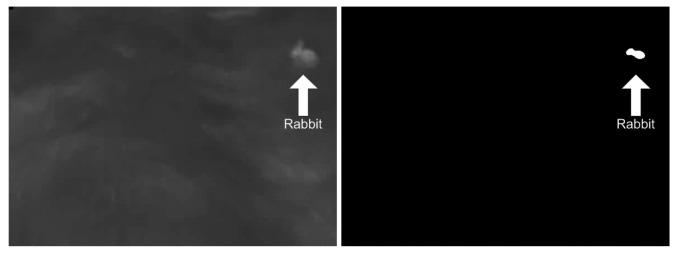
Example of binary image, where the rabbit is white (1) and the background is black (0). Figure 5 (left) shows the original image and Figure 5(right) shows the binary image where only the rabbit is visible.

The result is a binary image consisting of areas believed to be either an animal or background (Figure 5).

**Figure 6.**
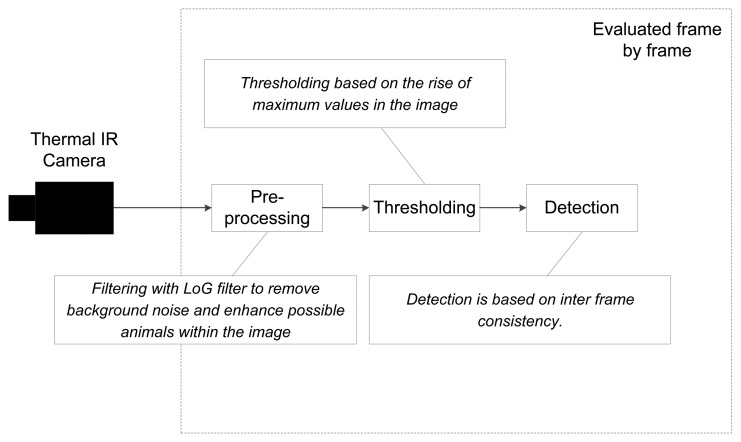
Flow of the image processing for the automatic detection of animals using thermal imaging.

To ensure robustness to false detections, it is required that an animal have to be detected within a given region in three consecutive frames (inter frame consistency), as the frame rate governs the maximum distance the animal is capable of moving from frame to frame. In Figure 6, a schematic presentation of the image processing algorithm for automatic animal detection is shown. We used this approach to test the suitability of using thermal imaging to automatically detect our study animals on the recorded videos. For comparison, we manually labelled the frames containing an animal and its position through visual inspection of the individual frames in the video recordings.

## 3. Results

The results of the automatic detection of our study animals at different driving speeds are expressed as the number of frames with true positive and false positive detections (Table 1).

The detection rate was almost 100% at all driving speeds (*i.e.*, true positives were obtained for most frames with an animal present), whereas in one case, the chicken at 15 km/h, one frame was erroneously classified as containing an animal (false positive).

In one test scenario, where the chicken was covered in dense grass, the system was not able to detect it until it was very close to the camera. In this case, the manual labelling was also challenging and was only possible on the basis of the frames recorded closest to the camera.

The choice of parameters could affect the detection of the animals in the first couple of frames of animal presence. The choice of parameters is based on the size of the animal, which is small when it first enters the frame, as the tractor was driving towards the animal. The effect of pre-processing therefore makes it difficult to detect the animal in this scenario as it would not be enhanced. This could explain why some algorithm fails to detect all the frames in some of the recorded scenarios.

## 4. Discussion

In view of the fact that visibility through the crop was low, the thermal imaging setup we used holds potential for detecting animals during grassland mowing operations. Throughout the different test scenarios, our study animals were detected with a high precision, although, as we expected, the detection rate of the chicken in the most dense grass cover was poor not only using thermal imaging but also by visual inspection of the images. The poor detection rate in this situation was probably a result of a combination of dense grass cover shielding the chicken and the insulative property of the feathers.

Even at the highest speeds, which closely resembled real life grass mowing conditions, the detection rate was nearly 100%. Therefore, we conclude that in grass land mowing the visibility through the crop is more important for the detection of animals than the working speed.

Our recordings were done on the same day with stable weather conditions throughout the experiment. Therefore, further experiments are needed to assess the capability of the system to detect animals under more challenging weather conditions. Since thermal imaging relies on the temperature differential between the animal and the crop, warm and sunny conditions (*i.e.*, the optimal for many farming operations) may reduce the detection of animals in the crop considerably. However, we consider the temperatures during the experiment to be within the range normally experienced during the grass cutting season in Denmark.

Although mowing operations in grassland are considered to be associated with higher risk of mortality than other harvesting operations, more experiments should be carried out to assess the suitability of thermal imaging to detect animals in other crop types, which may be different with respect to density and height. In this case, different camera position and angle to the crop should be tested to optimize detection in different crop types. In particular, we expect that a higher mounting of the camera, which will result in a more top-down view over the cutting area, will increase the detection rate in dense crops.

In our study, the field of view of the camera was approximately two metres and a distance to the crops of approximately 5 m, which is only a smaller part of the potential working width in mowing operations. Therefore, there is a need to increase the field of view, both in width and distance, which could be achieved by increasing the distance to the crop by different camera positioning, multiple cameras or other lens types. However, one should bear in mind that other lens types with a broader field of view (*i.e.*, fish-eye effect) may decrease the effective resolution of the thermal image, since pixels in the periphery inevitably will be stretched. The effect of this could be tested in further research. A system with multiple cameras requires stitching of images, although the performance from each camera would be comparable to the results presented in this paper.

More experiments could be done to investigate the above, and this is also part of ongoing research within automatic thermal detection.

Based on the methods described in this paper, a system capable of warning the farmer or scaring the animal in a non-audible fashion could be developed and is part of ongoing research.

## 5. Conclusions

Thermal imaging and digital imaging processing may be an important tool for the improvement of wildlife-friendly farming practices and offers as such a potential for reducing wildlife mortality in agriculture.

We conclude that the use of thermal imaging for automated detection of animals during mowing operations holds potential. Under most circumstances, detection rates were close to 100%, although dense crops may hamper the detection of animals.

## Figures and Tables

**Table 1.** Number of true and false positives for rabbits and chickens at different driving speeds.

	**True positives**	**False positives**	**Number of frames with animal present** ^a^	**Number of frames in video recording**
**Rabbit at 4 km/h**	21	0	22	156
**Rabbit at 8 km/h**	13	0	13	124
**Rabbit at 12 km/h**	7	0	7	133
**Rabbit at 15 km/h**	5	0	5	128
**Chicken at 5 km/h** ^b^	4	0	15	193
**Chicken at 5 km/h**	20	0	21	206
**Chicken at 10 km/h**	11	0	11	150
**Chicken at 15 km/h**	6	1	7	130

a Animal detected by means of visual inspection

b Dense grass cover
